# Multilevel modeling of technology use, student engagement, and fitness outcomes in physical education classes

**DOI:** 10.3389/fpsyg.2024.1458899

**Published:** 2024-10-24

**Authors:** Zhaohang Cui, Yifan Song, Xiaojuan Du

**Affiliations:** Department of Physical Education, Liaoning Normal University, Dalian, China

**Keywords:** technology use, student engagement, personal attributes, fitness outcomes, physical education

## Abstract

**Introduction:**

The integration of technology in educational settings, particularly in physical education, has shown potential in enhancing learning experiences and improving physical health outcomes. This study aims to investigate the effects of technology use on student engagement and fitness outcomes, considering the mediating role of student engagement and the moderating influence of personal attributes.

**Methods:**

Utilizing a time-lagged design, the research collected data from 513 Chinese undergraduate students (52% male and, 48% female) aged between 18 and 24 years over three waves using structured questionnaires rated on a 5-point Likert scale.

**Results:**

Results revealed a significant positive relationship between technology use and both student engagement (*β* = 0.68, *p* < 0.01) and fitness outcomes (*β* = 0.60, *p* < 0.01). Student engagement significantly mediated the relationship between technology use and fitness outcomes (*β* = 0.57, *p* < 0.01). Personal attributes moderated the effects of technology use on student engagement (*β* = 0.54, *p* < 0.01) and fitness outcomes (*β* = 0.52, *p* < 0.01), indicating varied benefits among students based on individual characteristics.

**Discussion:**

These findings highlight the importance of tailoring technological applications in physical education to individual needs, suggesting that personalized approaches can significantly enhance the effectiveness of technology in improving fitness and engagement.

## Introduction

The importance of technology use in physical education has been increasingly recognized in recent years (Niu, 2023), as educators seek innovative ways to enhance student engagement and improve fitness outcomes (Tang, 2023). Technology use refers to the integration of digital tools such as fitness trackers, mobile applications, and interactive software specifically designed for physical education (Zhamardiy et al., 2020). These tools aim to facilitate student participation, provide real-time feedback, and enhance physical activities, making them more engaging and personalized for students (Ferguson et al., 2022). A growing body of research illuminates the transformative potential of technology in educational settings (Deng and Yu, 2023; Fitria, 2023; Timotheou et al., 2023; Wang et al., 2023; Wijayanto et al., 2023), particularly within the realm of physical education (El-Tanahi et al., 2024; Wallace et al., 2023). For instance, Østerlie et al. (2023) have shown that incorporating digital tools like fitness trackers and interactive apps can significantly increase student participation by making activities more engaging and accessible. Furthermore, Arufe-Giráldez et al. (2023) highlights that technology not only facilitates a more interactive learning environment but also provides real-time feedback, enabling students to monitor their progress and adjust their efforts in physical activities. This evidence suggests that technology, when effectively integrated, can serve as a powerful catalyst for enhancing the educational experiences and physical health of students, making a compelling case for its broader adoption in physical education curricula.

While the integration of technology in physical education is often associated with enhancing learning environments and potentially increasing activity levels, there is a notable gap in direct empirical evidence linking technology use specifically to improved fitness outcomes in students. Current literature predominantly highlights the role of technology in facilitating interactive experiences and providing feedback mechanisms (Esteve-Mon et al., 2023), but its direct effect on the physical health improvements over time remains underexamined. This oversight in research highlights the need for a focused examination of how technology impacts actual fitness achievements. Understanding this relationship is essential for substantiating claims about the benefits of technology in physical education (Calderón et al., 2020) and for designing interventions that effectively utilize technology to boost physical fitness (Skarzhinskaya and Sarafanova, 2020), thereby fulfilling the broader educational objectives of health and wellness promotion in schools.

In addition, the study seeks to explore the underlying mediating mechanisms by examining student engagement, a critical component often proposed as a bridge between technology use and fitness outcomes. Student engagement in physical education is defined as the degree to which students are involved, motivated, and actively participating in physical activities within their educational programs (Leo et al., 2022). Student engagement refers to the degree to which students are motivated, involved, and actively participating in physical activities during their educational program (Leo et al., 2022). This concept includes behavioral, emotional, and cognitive components, reflecting how invested students are in the physical education process (Fredricks et al., 2004). While previous research has established that technology can enhance the learning environment and potentially increase activity levels (Calderón et al., 2020), there is a less explored area regarding how this engagement translates into actual fitness improvements. The research gap lies in understanding the mediating role of engagement: how it specifically acts to transform the enhanced interactions provided by technology into measurable fitness gains. This aspect of student engagement is crucial because it provides insight into the process by which technological tools can be leveraged to not only capture students’ interest but also to effectively improve their physical health outcomes. Addressing this gap allows for a more comprehensive understanding of the pathways through which technology influences physical education, offering valuable information for the design of more effective educational tools and strategies.

**Figure 1:**
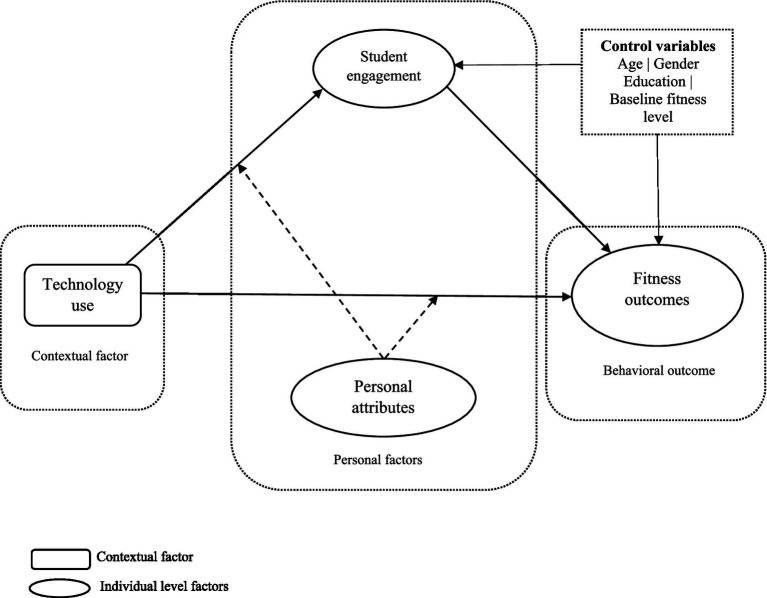
Proposed theoretical model.

Moreover, the study seeks to explore the boundary conditions by investigating the moderating influence of personal attributes on the relationship between technology use and fitness outcomes. Personal attributes, such as baseline fitness levels, motivational orientations, and individual attitudes towards physical education (Bishop and Durksen, 2020), can significantly influence how students respond to technological interventions in their classes. While existing research has highlighted the broad benefits of technology in educational settings (Alharthi, 2020), there is a notable lack of detailed understanding of how these effects might vary among individuals with different personal characteristics. This investigation into personal attributes as moderators is crucial because it could reveal that the effectiveness of technology in enhancing fitness outcomes is not uniform but instead depends on specific individual factors. By identifying these conditions, the study can provide more targeted insights and recommendations for customizing physical education programs to better meet the diverse needs of students, thereby optimizing the impact of technology on physical health improvements (Figure 1).

## Literature review

### Technology use and student engagement

The relationship between technology use and student engagement in physical education is hypothesized to be positively correlated. There is burgeoning evidence that modern students, often termed digital natives, are inherently stimulated by technology (Reddy and Bubonia, 2020; Wallace et al., 2023). When physical education classes incorporate elements such as fitness trackers, interactive apps, virtual reality, and online platforms (Zhamardiy et al., 2020), these tools can transform routine exercises into engaging, personalized experiences. This is further substantiated by Calabuig-Moreno et al. (2020) who argued that students who used fitness apps and trackers in physical education classes showed higher levels of participation and enthusiasm compared to those who did not. Similarly, Yu and Jee (2020) found that interactive technologies in physical education increased student engagement and improved overall physical fitness and health outcomes.

According to Yu (2023), technology facilitates various engaging features that cater to students’ interests and needs. For instance, wearable fitness trackers provide real-time feedback, allowing students to monitor their progress and set personal goals, which can be highly motivating (Ferguson et al., 2022). In addition, interactive apps offer personalized workout plans tailored to individual fitness levels, making activities more relevant and achievable (Zhamardiy et al., 2020). Further, gamification makes physical activities fun and competitive, appealing to students’ sense of play and competition (Ryan and Deci, 2000). These features are expected to boost engagement by making physical education classes more enjoyable and meaningful. In addition, self-determination theory (SDT) posits that engagement is driven by fulfilling autonomy, competence, and relatedness needs. Technology addresses these needs by providing control over learning (autonomy), immediate feedback (competence), and social interaction opportunities (relatedness) (Ryan and Deci, 2000). Empirical evidence supports that technology can foster a more intrinsically motivated and engaged student body.

### Mediating role of student engagement

The link between student engagement and fitness outcomes in physical education is essential to understand, as it is believed that higher levels of engagement can significantly boost physical health. When students are deeply involved in physical activities, they are more likely to put forth consistent effort, leading to better fitness results (Nuss et al., 2021). Research by Fredricks et al. (2004) highlights that students who show higher levels of engagement not only perform better academically but also exhibit enhanced physical performance.

According to Noetel et al. (2023), engagement in physical education involves active participation, emotional investment, and cognitive focus on physical tasks. These dimensions collectively ensure that students are not only physically active but also mentally and emotionally committed to improving their fitness. Anchored on the self-determination theory (SDT), when students feel autonomous, competent, and connected to others in their physical education classes, their engagement levels increase, which in turn enhances their physical outcomes (Ryan and Deci, 2000). Empirical evidence supports this notion. For instance, De Bruijn et al. (2020) found a positive correlation between student engagement and the amount of physical activity students performed, which directly improved their fitness levels. This study demonstrates that engaged students tend to participate more vigorously and frequently in physical activities, thereby gaining better fitness benefits. Similarly, Goodyear et al. (2023) showed that students who were more engaged in their physical education classes experienced notable improvements in their cardiovascular health and overall physical condition. This focus on engagement is crucial because it emphasizes the importance of creating a supportive and motivating environment in physical education. When students are engaged, they are more likely to enjoy physical activities and adopt healthier lifestyles, which can have lasting impacts on their fitness and well-being. Hence,

Subsequently, the integration of technology in physical education can significantly enhance student engagement, providing interactive and motivating experiences that capture students’ interest. This heightened engagement, driven by the use of digital tools such as fitness apps and wearable trackers (Goodyear et al., 2023), encourages more consistent and enthusiastic participation in physical activities. As students become more engaged through these technological interventions, they are likely to increase their physical activity levels (Leo et al., 2022), thereby improving their fitness outcomes. Thus, the use of technology not only makes physical education more engaging but also serves as a catalyst for better fitness achievements among students.

### Moderating role of personal attributes

Moreover, the study anticipates that personal attributes serve as boundary conditions between technology use and student engagement, as well as the indirect relationship between technology use and fitness outcomes through student engagement. Personal attributes, such as baseline fitness levels, motivational orientation, and individual attitudes toward physical education (Schmidt et al., 2020), are likely to influence how students interact with and benefit from technology in their physical education classes. For example, students with higher baseline fitness levels may be more inclined to use fitness trackers and apps effectively, resulting in greater engagement and subsequent fitness improvements. This notion aligns with findings from Pan (2020), who noted that individuals with a predisposition for physical activity tend to engage more with technological tools designed to enhance their workouts.

Motivational orientation is another critical personal attribute that can moderate the impact of technology on student engagement. According to Ryan and Deci (2000), students who are intrinsically motivated are more likely to engage deeply with educational content, including technology-enhanced physical education. When students find the use of technology enjoyable and aligned with their personal goals, their engagement levels are likely to increase (Lacka et al., 2021), leading to better fitness outcomes. This assumption is supported by the self-determination theory, which posits that autonomy, competence, and relatedness foster intrinsic motivation and engagement.

In addition, individual attitudes towards physical education also play a significant role in moderating these relationships. Students who have positive attitudes towards physical education are more likely to embrace technological tools and incorporate them into their fitness routines. As Alyoussef (2022) highlighted, students’ perceptions of the usefulness and ease of use of technology significantly impact their willingness to engage with it. When students perceive technology as beneficial and easy to use, their engagement increases, subsequently improving their fitness outcomes.

Furthermore, personal attributes may influence the indirect relationship between technology use and fitness outcomes through student engagement. For instance, a student with a high level of intrinsic motivation might not only engage more with the technology but also sustain this engagement over time, leading to more significant fitness improvements. Conversely, a student with lower motivation might initially engage with the technology but fail to maintain this engagement, resulting in less pronounced fitness outcomes.

The moderating role of personal attributes suggests that technology’s effectiveness in enhancing engagement and fitness outcomes is not uniform across all students. As Burton-Jones and Hubona (2005) noted, individual differences can significantly impact how students respond to educational interventions. By identifying and understanding these moderating factors, educators can tailor their approaches to better meet the diverse needs of their students, thereby maximizing the benefits of technology use in physical education.

## Hypotheses

Based on the aforementioned justifications, the study puts forth the following hypotheses:

*H1*: There is a positive relationship between technology use and student engagement.

*H2*: There is a positive relationship between student engagement and fitness outcomes.

*H3*: Student engagement mediates the relationship between technology use and fitness outcomes.

*H4*: The direct relationship between technology use and student engagement is moderated by personal attributes, such as higher levels of personal attributes underpin this association.

*H5*: The indirect relationship between technology use and fitness outcomes through student engagement is moderated by personal attributes, such as higher levels of personal attributes underpin this association.

## Methodology

### Sample and sampling

This study employed a quantitative methodology with a time-lagged design to investigate the impact of technology use in physical education classes on fitness outcomes, mediated by student engagement and moderated by personal attributes. The research unfolded across three waves spanning over 6 months, each strategically spaced to capture the evolving dynamics of the variables under consideration (Law et al., 2016).

The sampling strategy involved random selection to ensure a representative distribution of the population and to enhance the generalizability of the findings. A unique key was generated for each participant to match the responses across the three waves securely and accurately. This key was explained in a cover letter that accompanied each questionnaire, which also mentioned the study’s adherence to ethical guidelines in line with the Declaration of Helsinki and relevant institutions. Further, informed consent was obtained from all participants, ensuring confidentiality, voluntary participation. The cover letter reassured participants of their anonymity and the secure handling of their data, addressing any potential concerns about privacy. The letter also reiterated the study’s purpose, providing participants with a clear understanding of the importance of their consistent involvement across all three waves.

A total of 500 questionnaires were initially distributed to physical education students enrolled in Chinese universities in the first wave, which focused on collecting data on technology use, personal attributes, and demographic information. Of these, 543 responses were received due to the high participation rate; however, 30 responses were discarded due to incomplete or inconsistent answers, resulting in 513 valid questionnaires for the first phase.

In the second wave, aimed at measuring student engagement, 413 of the initial respondents were re-contacted, and 398 responses were successfully collected. The slight drop in participation was anticipated due to the longitudinal nature of the study.

The third and final wave targeted the measurement of fitness outcomes. All 398 participants from the second wave were invited to continue their participation, and 385 responded. After a detailed examination of these responses, 374 were deemed suitable for final analysis after consolidating the data and ensuring all entries met the study’s stringent quality criteria.

The methodological framework and data collection phases were designed to minimize biases and maximize the reliability and validity of the findings. The time-lagged design allowed for an effective examination of the causal relationships between the variables, providing robust insights into how technology use influences fitness outcomes through mechanisms of engagement, shaped by personal attributes.

### Demographic

The demographic profiles of the participants are as follows: the gender distribution was nearly balanced, with 52% of the participants identifying as male and 48% as female. Age was uniformly distributed across the educational years, reflecting participation from all academic levels: 18% were first-year students, 27% were second-year students, 28% were third-year students, and 27% were fourth-year students, ensuring a representation from each stage of undergraduate education. Concerning education, 21% of participants were first-year students, 24% were second-year students, 33% were third-year students, and 22% were fourth-year students. Lastly, baseline fitness level was categorized into three levels to gauge initial physical fitness: 33% of the participants reported a low fitness level, 34% reported a medium fitness level, and 33% reported a high fitness level.

### Control variables

In this study, several control variables were included to account for individual differences that might influence the relationships between technology use, student engagement, and fitness outcomes. The control variables—age, gender, education level, and baseline fitness level—were selected based on their potential impact on student responses to physical education interventions. Age was controlled for because physical fitness and engagement levels may vary with the developmental stage, as older students may demonstrate different physical readiness and motivation compared to younger students. Gender was also included to account for potential differences in how males and females engage with physical education and technology, as existing research suggests that males and females may have distinct preferences for fitness-related technology. Education level, or year of study, was controlled because students at different academic stages may have varying levels of engagement with physical education, influenced by their academic experiences and priorities. Baseline fitness level was particularly important, as students who begin with higher fitness levels may engage more actively and effectively with technology-enhanced physical education activities. Baseline fitness can affect a student’s motivation and capacity to respond to physical education interventions, making it a critical factor to control when examining fitness outcomes. By accounting for these variables, the study aimed to isolate the effects of technology use and student engagement on fitness outcomes and ensure that the results were not confounded by these individual characteristics.

### Measures

The research instruments for this study were designed to gather precise and reliable data from participants. Structured questionnaires were utilized as the primary data collection tool, featuring items designed to gauge perceptions and behaviors across several dimensions related to technology use, student engagement, fitness outcomes, and personal attributes in physical education settings. To ensure a standardized approach to responses, each item in the questionnaires was formatted using a 5-point Likert scale, ranging from 1 (“Strongly Disagree”) to 5 (“Strongly Agree”).

The technology use scale was adapted from existing validated instruments found in the work Kahveci (2010) and included five specific items. A sample item from this scale is, “I frequently use digital tools and apps in my physical education classes.” Similarly, the items measuring student engagement were developed by drawing insights from the foundational research by Fredricks et al. (2004), ensuring that the constructs were contextually relevant and grounded in educational psychology. The fitness outcomes scale included measures such as “My endurance has improved since the start of the semester,” reflecting physical development attributes typically impacted by engaged participation in physical education, adapted from Caspersen et al. (1985). Lastly, the personal attributes items were influenced by the work of Reeve (2013), and included elements such as motivation and discipline, crucial for understanding individual differences in physical education engagement and outcomes.

## Results

To examine the presence of common method variance (CMV), Harman’s single factor technique was employed. The observed variables were evaluated by exploratory factor analysis, employing an unrotated solution and constraining the number of factors to one for the purpose of examining single factor assessment. The finding reveals that the maximum variance explained by single factor was 30.342% of the total variance, falling below the threshold of 50%, suggesting that the dataset did not exhibit any concerns related to common method variance (CMV) (Podsakoff et al., 2003). While this is a commonly used method, it has limitations, particularly in its sensitivity to detect more subtle forms of method bias. As such, relying solely on Harman’s single-factor test may not provide a comprehensive assessment of CMV. To further strengthen the credibility of the results, future research could consider additional techniques for assessing CMV, such as the marker variable technique.

The study conducted confirmatory factor analyses (CFAs) using Mplus 7.4 to evaluate the discriminability of four key constructs: technology use, student engagement, fitness outcomes, and personal attributes. The analysis revealed that the proposed four-factor model demonstrated a good fit with the collected data, with a Chi-Square (χ^2^) value of 339.03 and 271 degrees of freedom, suggesting the model’s adequacy in representing the observed data. The Comparative Fit Index (CFI) and the Tucker-Lewis Index (TLI) both stood at 0.97, indicating a high degree of fit relative to the independent model. The Root Mean Square Error of Approximation (RMSEA) was 0.05 and the Standardized Root Mean Square Residual (SRMR) also at 0.05, both of which further support the model’s goodness of fit.

In comparison, alternative models demonstrated poorer fit indices: a three-factor model combining Technology Use and Student Engagement resulted in a χ^2^ of 755.24, CFI of 0.92, TLI of 0.92, RMSEA of 0.07, and SRMR of 0.09; a two-factor model that merged technology use, student engagement, and personal attributes showed a χ^2^ of 3230.97, CFI of 0.68, TLI of 0.65, RMSEA of 0.17, and SRMR of 0.21; and a one-factor model combining all four constructs had a χ^2^ of 6402.00, CFI of 0.31, TLI of 0.33, RMSEA of 0.27, and SRMR of 0.29. These comparisons underscore the superior performance of the four-factor model over the alternatives, affirming the distinctiveness of the constructs within the study.

Table 1 presents the descriptive statistics and correlations for the constructs of technology use, student engagement, fitness outcomes, and personal attributes measured at different times. The Cronbach’s alpha values, indicating internal consistency, are exceptionally high for all constructs, ranging from 0.90 to 0.97, which suggests that the scales used to measure these constructs are reliable.

**Table 1:** Correlations and descriptive statistics.

Construct	Mean	SD	1	2	3	4
1. Technology use (T1)	4.07	0.50	(0.90)	−0.49**	−0.20**	−0.60*
2. Student engagement (T2)	3.91	0.89	−0.23**	(0.94)	0.62**	0.54**
3. Fitness outcomes (T3)	3.83	0.85	−0.10*	0.20**	(0.97)	0.43**
4. Personal attributes (T1)	3.87	0.83	−0.29**	0.22**	0.21**	(0.96)

Values in diagonal report Cronbach alphas; values below and above diagonal reflect individual and contextual level correlations; T1, Time 1; T2, Time 2; T3, Time 3, **p < 0.01, *p < 0.05.

Technology use (T1) has a mean of 4.07 and a standard deviation of 0.50. It shows significant negative correlations with student engagement (T2) and personal attributes (T1), with coefficients of −0.49 and −0.29 respectively, indicating that higher technology use may be associated with lower student engagement and less favorable personal attributes. It also negatively correlates with fitness outcomes (T3) but to a lesser extent, with a coefficient of −0.20.

Student engagement (T2) is recorded with a mean of 3.91 and a standard deviation of 0.89. It positively correlates with fitness outcomes (T3) and personal attributes (T1), with coefficients of 0.62 and 0.22, suggesting that higher engagement is linked with better fitness outcomes and more favorable personal attributes.

Fitness outcomes (T3) has a mean of 3.83 and a standard deviation of 0.85, showing positive correlations with both student engagement (T2) and personal attributes (T1) with coefficients of 0.20 and 0.21, respectively. This indicates that improvements in fitness outcomes are moderately associated with higher engagement and more favorable personal attributes.

Personal attributes (T1) has a mean of 3.87 and a standard deviation of 0.83, and it shows a positive correlation with fitness outcomes (T3) and a negative correlation with technology use (T1), suggesting that favorable personal attributes might support better fitness outcomes while potentially being compromised by higher technology use.

Overall, the table reflects a complex interaction between technology use, student engagement, personal attributes, and fitness outcomes, highlighting the intricate dynamics within physical education settings.

The analysis of control variables provided additional insights into how individual differences affected student engagement and fitness outcomes. Age did not significantly influence either student engagement or fitness outcomes, indicating that the effects of technology use were consistent across different age groups. However, gender had a significant impact on fitness outcomes, with male students reporting slightly higher improvements in their fitness levels compared to females, although gender did not significantly affect student engagement. Education level showed a small but notable effect on student engagement, with students further along in their studies displaying higher engagement in physical education activities. This suggests that older students may have more developed skills or motivation to engage in physical education. In contrast, baseline fitness level was a strong predictor of fitness outcomes, as students who started with higher levels of fitness experienced greater improvements. However, baseline fitness had no significant effect on student engagement, suggesting that students across fitness levels were similarly engaged with the technology-enhanced activities.

In addition, Table 2 outlines the results of hypothesis testing for the study, examining the effects of various factors on student engagement and fitness outcomes at both individual and group levels.

**Table 2:** Hypothesis testing.

	Student engagement				Fitness outcomes			
	*Β*	se	*p*	95% CI	*Β*	se	*p*	95% CI
Within level								
Age	−0.04	0.03	0.79	[−0.15, 0.14]	0.02	0.02	0.70	[−0.05, 0.06]
Gender	−0.02	0.05	0.58	[−0.17, 0.12]	0.18**	0.07	0.01	[−0.32, −0.03]
Education	0.06*	0.05	0.02	[0.01, 0.13]	0.02	0.03	0.30	[−0.05, 0.08]
Baseline fitness level	0.03	0.03	0.21	[−0.02, 0.11]	0.03	0.02	0.48	[−0.04, 0.07]
Student engagement					0.07	0.04	0.15	[−0.03, 0.20]
Between level								
Technology use	0.68**	0.12	0.00	[0.39, 0.93]	0.60**	0.15	0.00	[0.30, 0.90]
Student engagement					0.60**	0.12	0.00	[0.31, 0.90]
Personal attributes	0.54**	0.03	0.00	[0.38, 0.70]	0.52**	0.11	0.00	[0.34, 0.69]
Technology use × personal attributes	0.57**	0.12	0.00	[0.38, 0.74]	0.63**	0.15	0.00	[0.48, 0.83]

**p < 0.01, *p < 0.05.

At the individual level, age showed no significant impact on student engagement or fitness outcomes, with beta coefficients near zero and high *p*-values, indicating a lack of strong influence. Gender also did not significantly affect student engagement, but it did positively impact fitness outcomes, suggesting that gender might play a role in how fitness is affected in the educational context. Education level had a small but significant positive effect on student engagement, suggesting that higher educational levels might boost engagement in physical activities. Baseline fitness level and student engagement, when tested as predictors, showed no significant influence on the fitness outcomes, although the directions of their effects were positive.

The results from Table 2 strongly support the hypothesis that technology use significantly enhances both student engagement and fitness outcomes in physical education classes. The positive impact of technology use is reflected in the substantial beta values for student engagement (*β* = 0.68, *p* < 0.01) and fitness outcomes (*β* = 0.60, *p* < 0.01), indicating that technology integration is crucial for improving these aspects of physical education.

Personal attributes also play a significant role in influencing both student engagement and fitness outcomes, as evidenced by positive beta values (*β* = 0.54 for engagement and *β* = 0.52 for fitness outcomes, both with *p* < 0.01). This finding suggests that individual characteristics can significantly contribute to how students engage and benefit from physical education programs.

**Figure 2:**
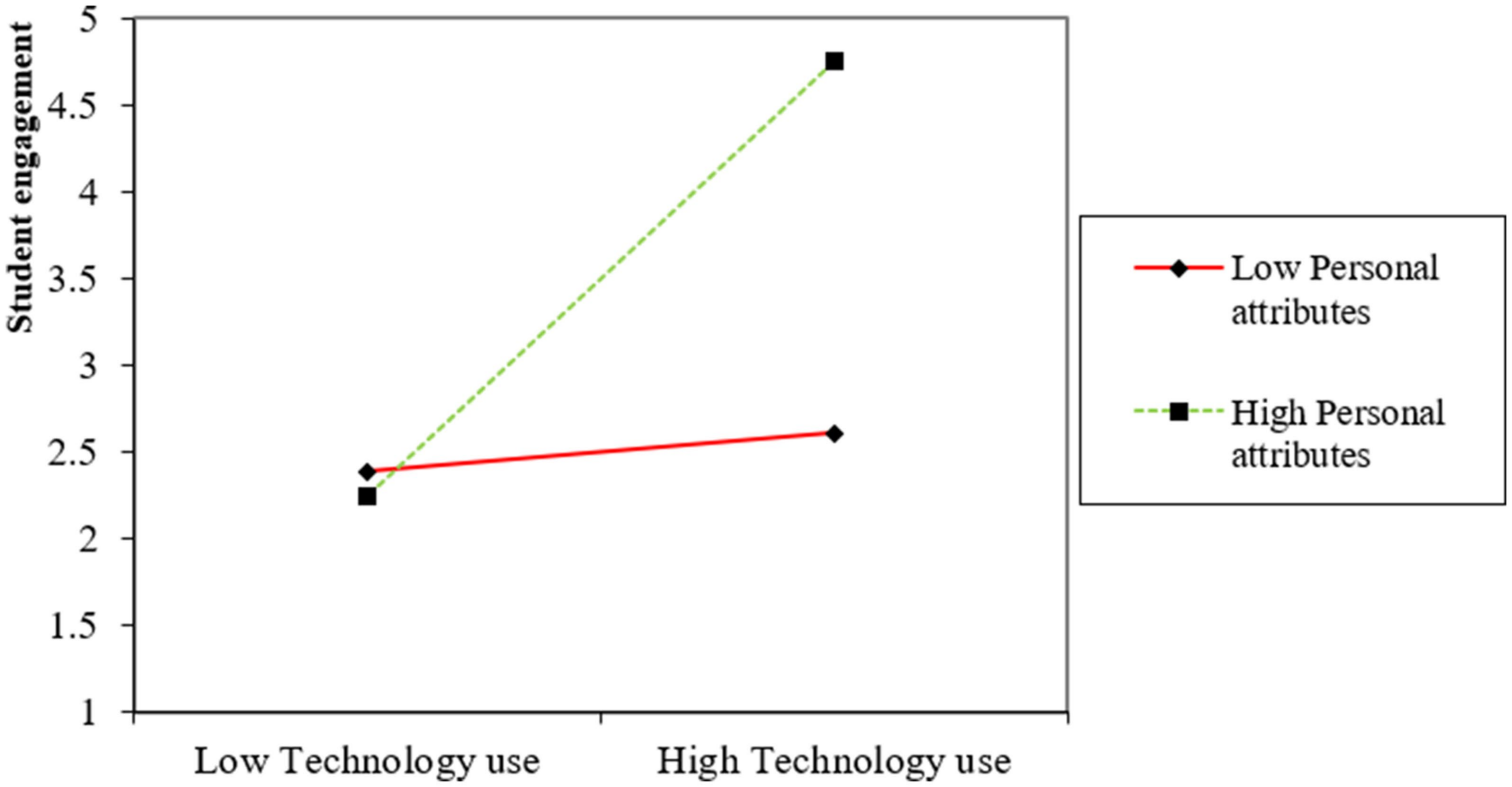
Moderation effect of personal attributes.

Furthermore, the interaction between technology use and personal attributes shows even stronger effects, with beta values of *β* = 0.57 for engagement and *β* = 0.63 for fitness outcomes (both *p* < 0.01). This interaction indicates that the benefits of technology use are enhanced when aligned with favorable personal attributes, leading to even greater improvements in engagement and fitness outcomes. The result of this analysis is also shown in the form of simple slope analysis in Figure 2.

Overall, these results highlight the critical importance of considering both technology use and personal attributes in designing effective physical education programs that can significantly enhance student engagement and fitness outcomes. The findings support the integration of tailored technological solutions that complement individual student characteristics to maximize educational and health benefits in physical education settings.

## Discussion

The study set out to explore the relationship between technology use in physical education and its impact on student engagement and fitness outcomes, while examining the mediating role of student engagement and the moderating effects of personal attributes. We successfully met these objectives, offering a nuanced understanding of how technology influences physical education.

First and foremost, technology use and fitness outcomes demonstrated a significant positive relationship, indicating that the incorporation of technology in physical education leads to improved physical health metrics among students. This aligns with findings from prior research such as Lai and Bower (2019) and (Yu, 2023), which suggest that technological tools can enhance the learning environment by providing interactive and engaging ways to participate in physical activities. These tools not only facilitate a more dynamic interaction with physical education content but also provide immediate feedback that can motivate students to push their physical limits (Lai and Bower, 2020).

Second, the role of student engagement as a mediator adds depth to our understanding. It suggests that technology’s impact on fitness outcomes is partially driven by how it increases student engagement. This is in line with theories like the Self-Determination Theory (Ryan and Deci, 2000), which emphasize the importance of engaging educational environments in enhancing intrinsic motivation, thereby improving learning and performance outcomes. In our study, the data indicated that higher levels of engagement, fostered by technology use, directly correlate with better fitness results, supporting the argument that engaged students are more likely to benefit physically from their educational experiences.

Moreover, the moderating role of personal attributes revealed that the benefits of technology in physical education are not uniformly experienced by all students. Instead, these benefits are more pronounced in students with specific characteristics such as higher baseline fitness levels or more positive attitudes towards physical education. This insight is crucial as it suggests that while technology can be a powerful tool, its effectiveness is contingent on individual differences (Chocarro et al., 2023). This finding encourages further investigation into personalized educational approaches that could maximize the effectiveness of technology-based interventions.

The moderated mediation analysis further demonstrated that the relationship between technology use and fitness outcomes through student engagement is significantly influenced by personal attributes. This complexity highlights the importance of considering individual student profiles when designing and implementing technology in physical education programs, ensuring that these interventions are tailored to meet diverse student needs and optimize educational outcomes.

In summary, the study contributes to the broader discourse on educational technology by demonstrating that while technology can significantly enhance physical education outcomes, the extent of its effectiveness depends substantially on how it interacts with student engagement and individual differences. This calls for a more personalized approach in the deployment of technology in educational settings, aiming to cater to the varied needs of students to fully harness the potential of technological advancements in education.

### Theoretical implications

The theoretical implications of this study are profound, contributing significantly to our understanding of the dynamics between technology use in educational settings, particularly in physical education, and its effects on student outcomes. These findings enrich the theoretical landscape in several key ways.

Firstly, the positive relationship between technology use and both student engagement and fitness outcomes support and extends existing educational theories that advocate for the integration of technology to enhance learning experiences. The findings affirm theories like the Technology Acceptance Model (TAM, Natasia et al., 2022), which posits that perceived usefulness and ease of use influence individuals’ acceptance and use of technology. By demonstrating that technology use in physical education can lead to improved fitness outcomes, this study provides empirical support for TAM in the context of physical education, suggesting that the benefits of technology are not only perceived but are also manifested in tangible improvements in student fitness levels.

Moreover, the mediation effect of student engagement between technology use and fitness outcomes offers a theoretical extension to the Self-Determination Theory (SDT, Ryan and Deci, 2000). According to SDT (Ryan and Deci, 2000), supportive environments that fulfill students’ needs for autonomy, competence, and relatedness can enhance motivation and engagement. The findings from this study suggest that technology acts as a catalyst in this environment, enhancing engagement by providing students with innovative and interactive ways to participate in physical activities, which in turn improves their physical health. This reinforces the importance of engagement as a central mechanism through which technology influences educational outcomes, emphasizing the need for educators to focus on how technology can foster an engaging learning environment.

Additionally, the moderation of these effects by personal attributes introduces a nuanced perspective to the discussion, aligning with the Differentiated Instruction (DI) theory (Griful-Freixenet et al., 2020) which advocates for educational practices that are responsive to individual learner differences. The moderating role of personal attributes, particularly baseline fitness levels and motivational orientation, provides key insights into how students interact with technology in physical education. Students with higher baseline fitness levels may find technology-enhanced activities more engaging because they are already physically prepared to participate more actively. Their prior experience with physical activity could make them more confident and comfortable using fitness apps and trackers, leading to greater engagement and improved fitness outcomes. On the other hand, students with lower baseline fitness levels may encounter more challenges when engaging with physical tasks, which could lower their overall engagement. However, personalized workout plans and real-time feedback provided by technology could still offer benefits to these students, as they can track their progress and work toward achievable goals. This suggests that tailored interventions might be necessary to maximize the engagement and fitness outcomes for students with lower fitness levels.

Motivational orientation also plays an important role in moderating these relationships. Students with a higher level of intrinsic motivation are likely to engage more deeply with technology-enhanced physical education, as it aligns with their personal goals for self-improvement and health. The autonomy and real-time feedback provided by the technology meet their intrinsic needs for competence and achievement, further enhancing their engagement and fitness outcomes. In contrast, students with lower motivation may require more external encouragement or simplified technological features to stay engaged. These findings emphasize the need for educators to personalize technology-based interventions in physical education, taking into account the unique characteristics of each student, such as their baseline fitness levels and motivational orientation. By doing so, educators can optimize the benefits of technology and create more effective learning experiences for all students.

### Practical implications

The practical implications of this study are significant for educators, policymakers, and curriculum designers, particularly in physical education. The findings provide actionable insights into how technology can be strategically utilized to enhance student engagement and improve fitness outcomes, informing several practical applications in educational settings.

The positive impact of technology use on student engagement and fitness outcomes suggests that schools and educational institutions should increase the integration of digital tools, such as fitness trackers, interactive apps, and virtual reality systems. These tools can make physical activities more engaging and measurable. Educators should be trained not only in the technical use of these tools but also in pedagogical strategies that leverage technology to enhance student motivation and participation.

Given the moderating role of personal attributes, it is essential for physical education programs to adopt personalized approaches. Assessing students’ baseline fitness levels, motivational orientations, and other personal characteristics at the beginning of courses can allow educators to tailor the use of technology accordingly. For example, students with lower fitness levels could benefit from technology-enhanced activities designed to gradually build their confidence and fitness.

These findings can also inform curriculum development, ensuring that technology is integrated in ways that align with educational goals. Curriculum designers should focus on structured lesson plans that incorporate technology for both instruction and feedback, providing students with a clear understanding of their progress and areas for improvement. Furthermore, the development of tailored interventions that consider individual student differences can further enhance engagement and outcomes.

In addition, the study suggests incorporating the taxonomy of teacher motivational behaviors (TMBs, Ahmadi et al., 2023). Using this classification of 57 behaviors related to autonomy, competence, and relatedness, educators can better support student motivation. For instance, offering choice in physical activities, providing individualized feedback, and fostering a supportive environment can enhance engagement and fitness outcomes.

At the policy level, the findings support the need for funding and resource allocation to expand technology integration in physical education. Policymakers can use this data to justify budget increases or to pilot programs that explore new forms of technology-enhanced physical education.

Lastly, schools should implement long-term monitoring and assessment strategies to evaluate the ongoing impact of technology on student engagement and fitness outcomes. This could include periodic surveys, fitness assessments, and feedback sessions, which would allow schools to continuously refine their approaches and maximize the benefits of technology in education.

By implementing these practical implications, educational institutions can significantly enhance the effectiveness of their physical education programs, leading to more engaged students and improved health outcomes.

## Limitations and directions for future research

While this study provides valuable insights into the use of technology in physical education, it also comes with certain limitations that warrant further reflection. First, the sample was limited to Chinese undergraduate students, which restricts the generalizability of the findings to other cultural or age demographics. Physical education practices, technology adoption, and engagement with fitness tools may vary significantly across different cultural contexts, making it important to replicate this research with more diverse populations in future studies. Furthermore, the age group of undergraduate students might limit the applicability of the findings to other educational levels, such as high school students or adult learners.

A second important limitation lies in the reliance on self-reported measures for technology use, student engagement, and fitness outcomes. Self-reported data may introduce biases, such as social desirability bias, where participants might overstate their engagement or fitness improvements. This method of data collection may also be prone to inaccuracies due to the subjective nature of self-assessment. Future studies could enhance data accuracy by incorporating more objective measures of fitness, such as standardized fitness tests or wearable fitness trackers, which could offer more reliable insights into the actual physical improvements achieved.

Additionally, the relatively short time frame of this study limits the ability to make conclusive statements about the long-term effects of technology use on fitness and engagement. The time-lagged design allowed for observation of short-term changes, but a longer-term longitudinal study would provide more robust evidence of how technology influences physical fitness and student engagement over time. A study with multiple follow-up points could also better assess the sustainability of the benefits associated with technology use in physical education.

The study also did not differentiate between various types of technology, which may have different impacts on student engagement and fitness outcomes. Different digital tools, such as fitness trackers, virtual reality systems, or mobile applications, may influence student engagement and outcomes in unique ways. Future research could explore the comparative effects of different types of technology to better understand which tools are most effective in enhancing student engagement and fitness in physical education settings.

Finally, while personal attributes were considered as moderating factors, other important variables, such as socio-economic status, technological literacy, or institutional support for technology use, were not included in the analysis. These factors could significantly impact the effectiveness of technology-enhanced physical education, and their inclusion in future studies could provide deeper insights into the conditions under which technology enhances educational outcomes.

Future research should address these limitations by expanding the study to include more diverse populations from different regions and educational levels, incorporating objective measures of fitness and engagement, extending the study’s duration, and exploring a wider range of moderating variables. These efforts would not only improve the theoretical frameworks underpinning this field but also refine the practical applications, leading to more personalized and effective educational strategies.

## Conclusion

The study highlights the significant positive impact of technology use on student engagement and fitness outcomes in physical education, emphasizing the potential of digital tools to enhance learning experiences and promote physical well-being. The findings reveal that technology’s effectiveness is mediated by student engagement and moderated by individual attributes, suggesting that personalized approaches in educational technology could maximize its benefits. While the study provides a solid foundation for integrating technology in physical education, future research should aim to address its limitations by expanding the participant base, refining measurement methods, and exploring long-term effects. By continuing to explore these areas, educators and policymakers can better harness the transformative potential of technology to enrich physical education programs across diverse educational settings.

## Data Availability

The raw data supporting the conclusions of this article will be made available by the authors, without undue reservation.

## References

[ref1] Ahmadi A. Noetel M. Parker P. Ryan R. M. Ntoumanis N. Reeve J.. (2023). A classification system for teachers’ motivational behaviors recommended in self-determination theory interventions. J. Educ. Psychol. 115, 1158–1176. doi: 10.1037/edu0000783

[ref2] Alharthi M. (2020). Students' attitudes toward the use of Technology in Online Courses. Int. J. Technol. Educ. 3, 14–23. doi: 10.46328/ijte.v3i1.18

[ref3] Alyoussef I. Y. (2022). Acceptance of a flipped classroom to improve university students’ learning: an empirical study on the TAM model and the unified theory of acceptance and use of technology (UTAUT). Heliyon 8:e12529. doi: 10.1016/j.heliyon.2022.e12529, PMID: 36619432 PMC9816777

[ref4] Arufe-Giráldez V. Sanmiguel-Rodríguez A. Ramos-Álvarez O. Navarro-Patón R. (2023). News of the pedagogical models in physical education—a quick review. Int. J. Environ. Res. Public Health 20:2586. doi: 10.3390/ijerph20032586, PMID: 36767953 PMC9916296

[ref5] Bishop M. Durksen T. L. (2020). What are the personal attributes a teacher needs to engage indigenous students effectively in the learning process? Reviewing the literature. Educ. Res. 62, 181–198. doi: 10.1080/00131881.2020.1755334

[ref6] Burton-Jones A. Hubona G. S. (2005). Individual differences and usage behavior: revisiting a technology acceptance model assumption. ACM SIGMIS Database 36, 58–77. doi: 10.1145/1066149.1066155

[ref7] Calabuig-Moreno F. González-Serrano M. H. Fombona J. Garcia-Tascon M. (2020). The emergence of technology in physical education: a general bibliometric analysis with a focus on virtual and augmented reality. Sustain. For. 12:2728. doi: 10.3390/su12072728

[ref8] Calderón A. Merono L. MacPhail A. (2020). A student-centred digital technology approach: the relationship between intrinsic motivation, learning climate and academic achievement of physical education pre-service teachers. Eur. Phys. Educ. Rev. 26, 241–262. doi: 10.1177/1356336X19850852

[ref9] Caspersen C. J. Powell K. E. Christenson G. M. (1985). Physical activity, exercise, and physical fitness: definitions and distinctions for health-related research. Public Health Rep. 100, 126–131, PMID: 3920711 PMC1424733

[ref10] Chocarro R. Cortiñas M. Marcos-Matás G. (2023). Teachers’ attitudes towards chatbots in education: a technology acceptance model approach considering the effect of social language, bot proactiveness, and users’ characteristics. Educ. Stud. 49, 295–313. doi: 10.1080/03055698.2020.1850426

[ref11] De Bruijn A. G. Kostons D. D. Van Der Fels I. M. Visscher C. Oosterlaan J. Hartman E.. (2020). Effects of aerobic and cognitively-engaging physical activity on academic skills: a cluster randomized controlled trial. J. Sports Sci. 38, 1806–1817. doi: 10.1080/02640414.2020.1756680, PMID: 32567975

[ref12] Deng X. Yu Z. (2023). A meta-analysis and systematic review of the effect of chatbot technology use in sustainable education. Sustain. For. 15:2940. doi: 10.3390/su15042940

[ref13] El-Tanahi N. Soliman M. Hady H. A. Alfrehat R. Faid R. Abdelmoneim M.. (2024). The effectiveness of gamification in physical education: a systematic review. Int. J. Educ. Math. Sci. Technol. 12, 406–417. doi: 10.46328/ijemst.4005

[ref14] Esteve-Mon F. M. Postigo-Fuentes A. Y. Castañeda L. (2023). A strategic approach of the crucial elements for the implementation of digital tools and processes in higher education. High. Educ. Q. 77, 558–573. doi: 10.1111/hequ.12411

[ref15] Ferguson T. Olds T. Curtis R. Blake H. Crozier A. J. Dankiw K.. (2022). Effectiveness of wearable activity trackers to increase physical activity and improve health: a systematic review of systematic reviews and meta-analyses. Lancet Digital Health 4, e615–e626. doi: 10.1016/S2589-7500(22)00111-X, PMID: 35868813

[ref16] Fitria T. N. (2023). Augmented reality (AR) and virtual reality (VR) technology in education: media of teaching and learning: a review. Int. J. Comput. Inform. Syst. 4, 14–25. doi: 10.15294/elt.v12i1.64069

[ref17] Fredricks J. A. Blumenfeld P. C. Paris A. H. (2004). School engagement: potential of the concept, state of the evidence. Rev. Educ. Res. 74, 59–109. doi: 10.3102/00346543074001059

[ref18] Goodyear V. A. Skinner B. McKeever J. Griffiths M. (2023). The influence of online physical activity interventions on children and young people’s engagement with physical activity: a systematic review. Phys. Educ. Sport Pedagog. 28, 94–108. doi: 10.1080/17408989.2021.1953459

[ref19] Griful-Freixenet J. Struyven K. Vantieghem W. Gheyssens E. (2020). Exploring the interrelationship between universal Design for Learning (UDL) and differentiated instruction (DI): a systematic review. Educ. Res. Rev. 29:100306. doi: 10.1016/j.edurev.2019.100306

[ref20] Kahveci M. (2010). Students' perceptions to use Technology for Learning: measurement integrity of the modified Fennema-Sherman attitudes scales. Turkish Online J. Educ. Technol. 9, 185–201. doi: 10.1080/09500690903127649

[ref21] Lacka E. Wong T. C. Haddoud M. Y. (2021). Can digital technologies improve students' efficiency? Exploring the role of virtual learning environment and social media use in higher education. Comput. Educ. 163:104099. doi: 10.1016/j.compedu.2020.104099

[ref22] Lai J. W. Bower M. (2019). How is the use of technology in education evaluated? A systematic review. Comput. Educ. 133, 27–42. doi: 10.1016/j.compedu.2019.01.010

[ref23] Lai J. W. Bower M. (2020). Evaluation of technology use in education: findings from a critical analysis of systematic literature reviews. J. Comput. Assist. Learn. 36, 241–259. doi: 10.1111/jcal.12412

[ref24] Law K. S. Wong C. S. Yan M. Huang G. (2016). Asian researchers should be more critical: the example of testing mediators using time-lagged data. Asia Pac. J. Manag. 33, 319–341. doi: 10.1007/s10490-015-9453-9

[ref25] Leo F. M. Mouratidis A. Pulido J. J. López-Gajardo M. A. Sánchez-Oliva D. (2022). Perceived teachers’ behavior and students’ engagement in physical education: the mediating role of basic psychological needs and self-determined motivation. Phys. Educ. Sport Pedagog. 27, 59–76. doi: 10.1080/17408989.2020.1850667

[ref26] Natasia S. R. Wiranti Y. T. Parastika A. (2022). Acceptance analysis of NUADU as e-learning platform using the technology acceptance model (TAM) approach. Procedia Comput. Sci. 197, 512–520. doi: 10.1016/j.procs.2021.12.168

[ref27] Niu Y. (2023). Integrated physical education and medicine in general physical education at universities in the age of educational technologies. BMC Med. Educ. 23:466. doi: 10.1186/s12909-023-04440-9, PMID: 37349726 PMC10286391

[ref28] Noetel M. Parker P. Dicke T. Beauchamp M. R. Ntoumanis N. Hulteen R. M.. (2023). Prediction versus explanation in educational psychology: a cross-theoretical approach to using teacher behaviour to predict student engagement in physical education. Educ. Psychol. Rev. 35:73. doi: 10.1007/s10648-023-09786-6

[ref29] Nuss K. Moore K. Nelson T. Li K. (2021). Effects of motivational interviewing and wearable fitness trackers on motivation and physical activity: a systematic review. Am. J. Health Promot. 35, 226–235. doi: 10.1177/0890117120939030, PMID: 32662277

[ref30] Østerlie O. Sargent J. Killian C. Garcia-Jaen M. García-Martínez S. Ferriz-Valero A. (2023). Flipped learning in physical education: a scoping review. Eur. Phys. Educ. Rev. 29, 125–144. doi: 10.1177/1356336X221120939

[ref31] Pan X. (2020). Technology acceptance, technological self-efficacy, and attitude toward technology-based self-directed learning: learning motivation as a mediator. Front. Psychol. 11:564294. doi: 10.3389/fpsyg.2020.564294, PMID: 33192838 PMC7653185

[ref001] Podsakoff P. M. MacKenzie S. B. Lee J. Y. Podsakoff N. P. (2003). Common method biases in behavioral research: a critical review of the literature and recommended remedies. J Appl Psychol. 88:879. 14516251 10.1037/0021-9010.88.5.879

[ref32] Reddy S. L. Bubonia J. (2020). Technology in education: learning opportunities for teachers and students. J. Family Consumer Sci. 112, 46–50. doi: 10.14307/JFCS112.1.46

[ref33] Reeve J. (2013). How students create motivationally supportive learning environments for themselves: the concept of agentic engagement. J. Educ. Psychol. 105, 579–595. doi: 10.1037/a0032690

[ref34] Ryan R. M. Deci E. L. (2000). Intrinsic and extrinsic motivations: classic definitions and new directions. Contemp. Educ. Psychol. 25, 54–67. doi: 10.1006/ceps.1999.1020, PMID: 10620381

[ref35] Schmidt S. K. Reinboth M. S. Resaland G. K. Bratland-Sanda S. (2020). Changes in physical activity, physical fitness and well-being following a school-based health promotion program in a Norwegian region with a poor public health profile: a non-randomized controlled study in early adolescents. Int. J. Environ. Res. Public Health 17:896. doi: 10.3390/ijerph17030896, PMID: 32023995 PMC7037455

[ref36] Skarzhinskaya E. N. Sarafanova E. V. (2020). “Digital technologies in physical culture and sports education” in First international Volga region conference on economics, humanities and sports (FICEHS 2019) (Atlantis Press), 805–807.

[ref37] Tang H. (2023). RETRACTED: applied research of VR technology in physical education. Int. J. Electrical Engin. Educ. 60, 2652–2664. doi: 10.1177/00207209211007774

[ref38] Timotheou S. Miliou O. Dimitriadis Y. Sobrino S. V. Giannoutsou N. Cachia R.. (2023). Impacts of digital technologies on education and factors influencing schools' digital capacity and transformation: a literature review. Educ. Inf. Technol. 28, 6695–6726. doi: 10.1007/s10639-022-11431-8, PMID: 36465416 PMC9684747

[ref39] Wallace J. Scanlon D. Calderón A. (2023). Digital technology and teacher digital competency in physical education: a holistic view of teacher and student perspectives. Curric. Stud. Health Physical Educ. 14, 271–287. doi: 10.1080/25742981.2022.2106881

[ref40] Wang C. Omar Dev R. D. Soh K. G. Mohd Nasirudddin N. J. Yuan Y. Ji X. (2023). Blended learning in physical education: a systematic review. Front. Public Health 11:1073423. doi: 10.3389/fpubh.2023.1073423, PMID: 36969628 PMC10034186

[ref41] Wijayanto P. W. Thamrin H. M. Haetami A. Mustoip S. Oktiawati U. Y. (2023). The potential of metaverse technology in education as a transformation of learning media in Indonesia. Jurnal Kependidikan 9, 396–407. doi: 10.33394/jk.v9i2.7395

[ref42] Yu Z. (2023). A meta-analysis of the effect of virtual reality technology use in education. Interact. Learn. Environ. 31, 4956–4976. doi: 10.1080/10494820.2021.1989466

[ref43] Yu J. Jee Y. (2020). Analysis of online classes in physical education during the COVID-19 pandemic. Educ. Sci. 11:3. doi: 10.3390/educsci11010003

[ref44] Zhamardiy V. Griban G. Shkola O. Fomenko O. Khrystenko D. Dikhtiarenko Z.. (2020). Methodical system of using fitness technologies in physical education of students. Int. J. Appl. Exerc. Physiol. 9, 27–34.

