# Supplementary material for: Multilevel modeling of technology use, student engagement, and fitness outcomes in physical education classes
Source: Front Psychol. 2024 Oct 24;15:1458899. doi: 10.3389/fpsyg.2024.1458899 (PMC11540672; doi:10.3389/fpsyg.2024.1458899)
Supplement: Supplementary file 1 [file Table_1.DOCX]

**Technology use**

1. "I frequently use digital tools and apps in my physical education classes."
2. "Technology in physical education helps me understand exercises better."
3. "I rely on wearable technology to monitor my physical activities during classes."
4. "Using technology motivates me to participate more actively in physical education."
5. "I find the technology used in our classes easy to interact with."

**Student engagement**

1. "I am attentive and focused during physical education classes."
2. "I look forward to participating in physical education classes."
3. "I actively participate in all physical activities required in class."
4. "I feel involved and included in the physical education activities."

**Fitness outcomes**

1. "My endurance has improved since the start of the semester."
2. "I have noticed an increase in my overall strength."
3. "My flexibility has improved due to the physical education classes."
4. "I am quicker and more agile than I was at the beginning of the course."
5. "My physical fitness has generally improved over the past semester."

**Personal attributes**

1. "I generally have a positive attitude towards physical education."
2. "I consider myself motivated when it comes to engaging in physical activities."
3. "I am disciplined in following the routines set in my physical education classes."
4. "I am resilient and persistent in overcoming physical challenges."
5. "I frequently set personal fitness goals."
6. "I believe in the importance of maintaining physical health."
